# Characteristics, Management, and Outcomes of Community-Acquired Pneumonia Due to Human Rhinovirus—A Retrospective Study

**DOI:** 10.1155/2022/1349994

**Published:** 2022-12-09

**Authors:** Ibrahim Bahabri, Abdulaziz Abdulaal, Thamer Alanazi, Sultan Alenazy, Yasser Alrumih, Rakan Alqahtani, Mohammad Bosaeed, Hasan M. Al-Dorzi

**Affiliations:** ^1^College of Medicine-Riyadh, King Saud Bin Abdulaziz University for Health Sciences, King Abdullah International Medical Research Center, Department of Medicine, King Abdulaziz Medical City, Ministry of National Guard Health Affairs, Riyadh, Saudi Arabia; ^2^College of Medicine-Riyadh, King Saud Bin Abdulaziz University for Health Sciences, King Abdullah International Medical Research Center, Intensive Care Department, King Abdulaziz Medical City, Ministry of National Guard Health Affairs, Riyadh, Saudi Arabia

## Abstract

**Introduction:**

Human rhinovirus (HRV) can lead to a variety of respiratory illnesses; it is also an uncommon cause of community-acquired pneumonia (CAP). We described the characteristics and outcomes of patients hospitalized for CAP due to HRV.

**Methods:**

We retrospectively studied consecutive adult patients admitted to King Abdulaziz Medical City-Riyadh with CAP due to HRV between 2016 and 2019. The diagnosis was made by respiratory multiplex PCR within 48 hours of hospitalization. We compared patients requiring ICU admission to those who did not.

**Results:**

One-hundred-and-six patients were studied (peak hospitalization between November and January, median age 71.5 years, hypertension 59%, diabetes 50%, and chronic respiratory disease 44.3%); 16 (15.1%) patients required ICU admission. The median pneumonia severity index score (PSI) was 107, with no significant difference between ICU and nonICU patients. ICU patients had a higher prevalence of tachypnea (62.5% vs. 26.7%, *p*=0.005), hemoptysis (12.5% vs 0%, *p*=0.001), and lymphopenia (71.4% vs 26.3%, *p*=0.01). Chest X-ray on presentation showed bilateral infiltrates in 47/101 (46.5%) patients and unilateral infiltrates in 26/101 (25.7%) patients. Systemic corticosteroids were used in 54.7% of patients (the median initial dose was 120 mg of prednisone equivalent and was higher in nonICU patients). Most (69.2%) ICU patients received mechanical ventilation (median duration of 8 days). Bacterial coinfection (6.6%) and superinfection (3.8%) were rare. The overall hospital mortality was 9.4% (higher for ICU patients: 37.5% vs. 4.4%, *p* < 0.001).

**Conclusions:**

Most patients with CAP due to HRV were elderly and had significant comorbidities. ICU admission was required in almost one in six patients and was associated with higher mortality.

## 1. Introduction

Community-acquired pneumonia (CAP) is a common cause of sepsis [1]. It represents a significant burden on the healthcare system, with approximately 650 per 100,000 population getting hospitalized with CAP every year in the United States, 100,000 annual deaths during hospitalization, and almost one-third of patients hospitalized with CAP dying within one year [2]. A considerable number of cases are severe enough to require intensive management, with 13–22% of patients requiring ICU admission and 20% of patients getting readmitted within 30 days [3].

Viral etiologies of CAP have often gone unidentified primarily due to limitations of diagnostic tests such as cell culture, antigen assays, and serology. Recent advancement in diagnostic testing, particularly the more sensitive polymerase chain reaction (PCR), has considerably increased the rate of detection of viruses [4, 5], which have been implicated in 30–40% of adult cases of CAP [6–8]. One systematic review and meta-analysis to identify respiratory viruses in adult patients with CAP in Europe found that the most common viruses were influenza A/B (9%), human rhinovirus (HRV) (5%), human coronavirus (4%), human parainfluenza virus (3%), human metapneumovirus (2%), respiratory syncytial virus (2%), and adenovirus (1%) [6]. Another systemic review found that in studies that used respiratory viral PCR routinely, HRV was detected in 4.1–11.5% of patients and was the second most common virus after influenza viruses (6.2–13.7%) [8]. HRV is notorious as a cause of the common cold [9]. However, it can cause more serious diseases among the elderly and immunocompromised [9]. A study of an outbreak in a long-term facility found that HRV infected 100% of its 56 residents and 26 of its healthcare providers [10]. The mortality among the older residents was 21%, compared to 0% among the healthcare providers who predominantly had a milder upper respiratory tract illness [10]. In another study of 1198 hospital admissions for acute respiratory illnesses, 45 patients had HRV infections with infected children likely developing bronchiolitis or pneumonia, young adults typically presenting with asthma exacerbation, and older immunocompromised patients presenting with pneumonia [11]. Other studies showed that HRV could lead to severe acute respiratory infections and acute respiratory failure [12–14]. One study from Korea that included 64 patients with severe CAP requiring ICU admission identified respiratory viruses by respiratory multiplex PCR in 26 (40.6%) patients(respiratory syncytial virus in 7, influenza in 6, and HRV in 4 patients) [14]. A study from Finland evaluated 49 patients with severe CAP requiring mechanical ventilation and found that HRV was the most common viral etiology (15 patients) [15]. Another study from Chile showed that HRV-related admissions (*N* = 32) represented 23.7% of hospitalization due to severe acute respiratory infections among adults, second only to influenza (37.8%) [12].

Data from Middle Eastern countries about pneumonia due to viruses other than influenza and coronavirus is scarce. A study from Prince Sultan Military Medical City in Riyadh that evaluated viral pneumonia due to influenza or coronavirus in 448 patients, with influenza A (nonH1N1)/influenza B affecting 216 patients (48.2%), H1N1 influenza 150 patients (33.5%), and Middle East respiratory syndrome (MERS) coronavirus 82 patients (18.3%) [16]. The objectives of this study were to characterize the patients with CAP due to HRV and determine their outcomes.

## 2. Materials and Methods

### 2.1. Patients and Settings

This was a retrospective cohort study of adult patients hospitalized with CAP due to HRV in King Abdulaziz Medical City, a 1400-bed tertiary-care hospital in Riyadh, Saudi Arabia, between January 1, 2016, and December 31, 2019. We excluded patients younger than 14 years and those who had HRV detected more than 48 hours after hospital admission. The Institutional Review Board of the Ministry of National Guard Health Affairs approved the study. Management of patients was according to the treating medical teams.

### 2.2. Microbiological Diagnosis

The diagnosis of CAP due to HRV was established by having symptoms and signs of CAP and detection of HRV by respiratory real-time multiplex PCR assays. Acceptable samples were sputum, endotracheal aspirate, and bronchoalveolar lavage fluid obtained within 48 hours of admission.

### 2.3. Data Collection

The list of patients who had a positive respiratory multiplex PCR or a positive influenza A/B PCR within the study period was obtained from the hospital's Microbiology Laboratory. For patients eligible for this study, we collected data on demographics, prehospitalization functional status, comorbid conditions, the month of hospitalization, presenting symptoms and signs (including laboratory and radiologic data), pneumonia severity index [17], presence of respiratory bacterial coinfection (growth of bacteria from a respiratory specimen within 48 hours of admission), management (antimicrobial and antiviral therapy, use of corticosteroids and dose, ICU admission, intubation and mechanical ventilation, and use of vasopressors). The primary outcome was hospital mortality. Secondary outcomes included the occurrence of hospital-acquired respiratory bacterial superinfection (growth of bacteria from a respiratory specimen after 48 hours of admission), duration of mechanical ventilation, the performance of tracheostomy, length of stay in the ICU and hospital, and ICU mortality.

### 2.4. Statistical Analysis

The patients were categorized into two groups depending on ICU admission. Baseline demographics, clinical characteristics, laboratory values, management aspects, and outcomes were presented as medians with interquartile range (for continuous variables) or frequencies with percentages (for categorical variables). Continuous variables were assessed for normality of distribution and were compared using the Student *t*-test or Mann–Whitney *U* test, as appropriate. Categorical variables were compared using the Chi-square test or Fisher's exact test, as appropriate. All statistical tests were considered significant at *α* level less than 0.05. Statistical analysis was performed using SPSS (SPSS Inc., SPSS for Windows, version 16.0. Chicago, IL, SPSS Inc.).

## 3. Results

### 3.1. Baseline Characteristics and Presenting Symptoms and Signs

**Figure 1:**
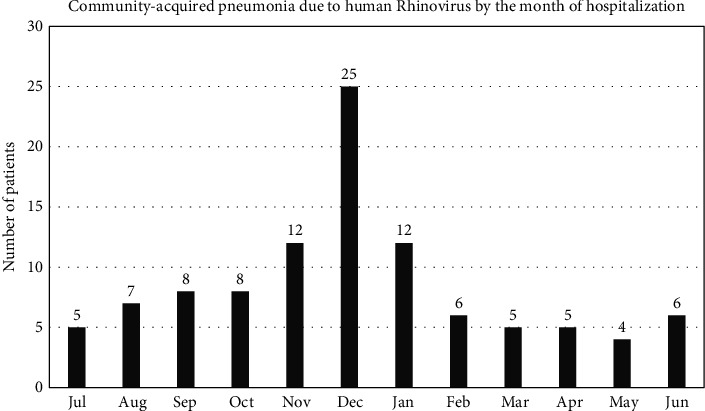
Distribution of community-acquired pneumonia due to human rhinovirus according to the month of hospital admission. Admissions occurred perennially, with a peak from November to January.

During the study period (48 months), 982 hospitalized patients had respiratory multiplex PCR. Influenza A/B virus was detected in 193 patients (73 by the respiratory multiplex PCR and 120 by the specific influenza PCR). HRV was detected in 106 patients, 10.8% of the respiratory multiplex PCR followed by human coronavirus (92 patients, 9.4%), respiratory syncytial virus (80 patients, 8.1%), and parainfluenza virus (41 patients, 4.2%). Hospitalizations for CAP due to HRV occurred perennially, with a peak number of admissions in the months of November to January, during which 49/106 (46.2%) were admitted (Figure 1). The demographics and comorbidities of patients in this cohort are presented in Table 1. In general, the patients were elderly, with a median age of 71.5 years, and had multiple comorbidities. The most common comorbidities in our population were hypertension (59%), followed by diabetes (50%). Nine patients (8.5%) had an immunocompromised state. The most common presenting symptom was shortness of breath (90.6%). The cough was most productive (66 patients presented with purulent cough compared with 25 patients with dry cough). For the inflammatory/infectious markers on admission, leukocytosis >15 × 10^9^/L was found in 18.9%, C-reactive protein >40 mg/L in 53.3%, and procalcitonin >0.5 ng/ml in 29.0%.

Sixteen patients (15.1%) were admitted to the ICU. There were no significant differences in demographics, comorbidities, and pneumonia severity index between ICU and nonICU patients (Table 1). Compared with patients not admitted to the ICU, ICU patients presented with more tachypnea (respiratory rate >30/min in 62.5% versus 32.1%, *p* = 0.005) and hemoptysis and had a higher median neutrophil percentage (86.4% versus 74% for, *p* = 0.02), lower median lymphocyte percentage (5.7% versus 13.1%, *p* = 0.01), more lymphopenia and higher neutrophil-to-lymphocyte ratio (median of 10.4 versus 5.2). No significant differences were found in any of the other assessed laboratory tests, including inflammatory/infectious markers such as white blood cells, C-reactive protein, and procalcitonin (Table 1). ICU patients tended to have more bilateral lung infiltrates. The prevalence of respiratory bacterial coinfection was similar in ICU and nonICU patients.

### 3.2. Management of Patients

Key management aspects are shown in Table 2. Antibiotics were provided for the vast majority (93.3%) of patients. Bacterial coinfection was diagnosed in 7 (6.6%) patients and superinfection in 5 (4.7%). The most widely used initial antibiotic regimen in nonICU patients was a combination of beta-lactam and azithromycin (40 patients, 44%). For ICU patients, the most widely used initial regimen was an antipseudomonal beta-lactam and azithromycin (10 patients, 62%). Oseltamivir was used initially in 73 out of 106 patients (68.9%), with no significant difference between ICU and nonICU patients. Most patients (58/106, 54.7%) received systemic corticosteroids with no significant difference between ICU and nonICU patients. However, patients who did not require ICU admission were given a significantly higher initial corticosteroid dose (120 mg of prednisone or equivalent daily) compared with patients who required ICU admission (50 mg of prednisone or equivalent daily, *p* = 0.02). The use of corticosteroids was more common in patients with chronic respiratory disease (32/47 (68.1%) patients versus 26/59 (44.1%) patients without chronic respiratory disease, *p* = 0.01).

Most patients (69.2%) admitted to the ICU received intubation and mechanical ventilation. More than one-third (37.5%) had shock requiring treatment with vasopressors.

### 3.3. Outcomes of Patients

**Figure 2:**
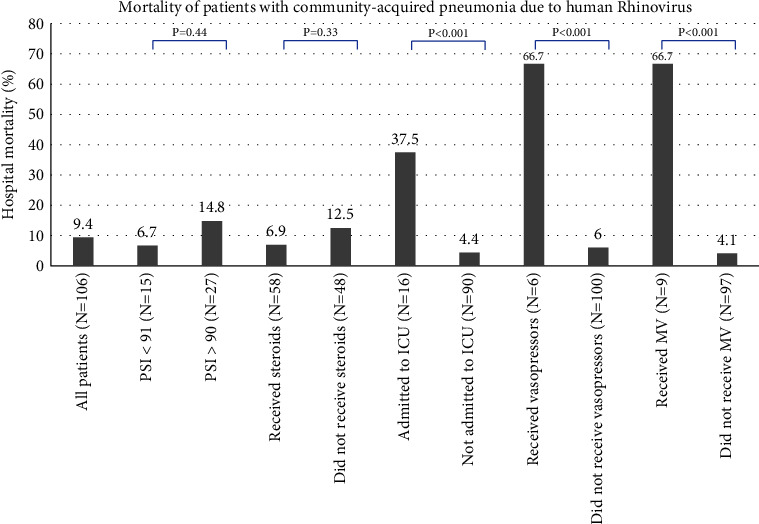
Hospital mortality of patients with community-acquired pneumonia due to human rhinovirus is categorized by important clinical exposures. ICU: intensive care unit, PSI: pneumonia severity index.

The outcomes of patients are described in Table 3. The overall mortality of the cohort was 9.4%.  Figure 2 describes the hospital mortality of patients categorized by important clinical exposures. The mortality of patients who received systemic corticosteroids was 4/58 (6.9%) compared with 6/48 (12.5%) for those who did receive corticosteroids (*p*=0.33). Patients who were admitted to the ICU, treated with vasopressors for shock, or given mechanical ventilation for acute respiratory failure had significantly higher mortality than those who were not (*p* < 0.001).

Bacterial superinfection occurred in only four (3.8%) patients. The median length of hospital stay was longer for ICU patients (median of 16.5 days versus 5 days for nonICU patients, *p* < 0.001). The median duration of mechanical ventilation was 8 days with only one patient (1.2%) having a tracheostomy.

## 4. Discussion

In this study, we characterized the patients with CAP due to HRV and assessed their outcomes. We found that hospitalizations peaked in the cold months (November to January); patients were elderly and commonly had comorbidities; 15.1% of patients were admitted to the ICU with mechanical ventilation provided in almost one in ten patients; hospital mortality was 9.4% and was significantly higher in patients who were admitted to the ICU.

HRV infection frequently involves the respiratory tract causing various respiratory illnesses, such as the common cold, exacerbation of asthma or chronic obstructive pulmonary disease, and pneumonia [18, 19]. There are multiple risk factors for developing severe acute respiratory illness due to HRV. In our study, HRV was the second most prevalent virus causing CAP requiring hospitalization after influenza A/B with most affected patients being elderly with a high prevalence of comorbidities such as diabetes, chronic cardiovascular disease, chronic respiratory disease, and immunocompromised state. HRV can cause a severe acute respiratory illness usually in older people with comorbidities and in those with an immunocompromised state [10–12, 20, 21]. One study found that when compared with patients with H1N1 influenza (*n* = 99), HRV-infected patients (*n* = 62) were more likely to be diabetic (24.2% versus 9.1%; *p*=0.01) and immunocompromised (27.4% versus 10.1%; *p* < 0.01) [21]. A high prevalence of diabetes and hypertension was also noted in a study from Saudi Arabia that evaluated patients with influenza viruses and MERS-CoV [16]. We found that 26.4% of our patients were bedbound before hospitalization. This suggests that poor functional status may be a risk factor for hospitalization after an HRV infection.

The clinical manifestations of HRV pneumonia in our study were similar to those reported in other studies. Cough and shortness of breath were the most prevalent symptoms with 87.7% of patients having a cough and 90.6% having shortness of breath in a study [21]. Purulent cough being more common than dry cough in our patients might go against the traditional doctrine of sputum production pointing more towards a bacterial etiology, but this finding is consistent with other studies on HRV pneumonia [10, 13, 20]. Unlike the study by Wang et al., where the majority of patients (85%) had a moderate to high-grade fever [13], less than half (38.7%) of our patients had fever in their history or on presentation. Two other studies showed that less than half of the patients admitted with HRV had fever at the time of admission [11, 12], suggesting that the lack of fever would not be a reliable indicator to rule out HRV pneumonia. The persistence of symptoms has been linked to viral shedding and infectiousness, which need to be recognized and considered for infection prevention and control purposes [22, 23]. The median pneumonia severity index in our cohort was 107, which is close to that observed in other studies [24]. We observed neutrophilic leukocytosis with a minority of patients having lymphopenia. Other studies found no significant leukocytosis and a high prevalence of lymphopenia [12, 21]. Lymphopenia can be a marker of more severe viral pneumonia [25] and was more common in patients admitted to the ICU in our study. In the current study, bacterial coinfection was present in only 6.6% of patients. This rate is lower than what was reported in other studies, where bacterial/viral coinfection was found in 25–35% of patients [8]. Bilateral lung infiltrates were commonly seen in our study, which is a typical pattern in viral pneumonia [11, 12, 16, 21]. In our study, 16 patients (15.1%) had a severe illness and were admitted to the ICU. Similar rates were observed for viral pneumonia in the study by Al-Baadani et al. (13.3%) and for HRV in the study by Fica et al. (11.3%) [12, 16]. In our study, 8.7% of patients developed respiratory failure requiring mechanical ventilation. Higher rates of mechanical ventilation were observed in other studies ranging from 25% to 65% [11, 13].

Treatment of HRV infection is mainly supportive [9]. In our study, 93.3% of patients were given antibiotics, as is the standard for the treatment of patients admitted with a working diagnosis of CAP [11–13]. The majority of patients in our study (68.9%) received oseltamivir on admission. This is commonly practiced in the empirical treatment of CAP [12], especially since most of our patients were admitted in the winter time, the peak time for influenza infection in Saudi Arabia [26]. Additionally, 58 out of 106 (54.7%) patients in the current study received systemic corticosteroids. Most (32/58, 60.3%) of these patients had chronic respiratory disease, including asthma and chronic obstructive pulmonary disease. HRV is known to cause exacerbations of asthma and chronic obstructive pulmonary disease [18, 19], which are usually treated with systemic corticosteroids. Additionally, HRV may have caused a wheezing illness in other patients and so led to corticosteroid use.

In our study, the hospital mortality of patients with CAP due to HRV was 9.4%, similar to that reported by Fica et al. (12.5%) [12] and by Al-Baadani et al. (13.8%) [16] but lower than the 28.6% mortality reported by Kraft et al. [21]. The mortality was significantly higher in patients who were admitted to the ICU (6 out of 16 [37.5%] patients). One study found that the mortality of patients admitted to the ICU with viral pneumonia was close to 25% [14]. The median length of stay in the hospital was 5 days, which is similar to the findings of other studies [11, 12]. Patients admitted to the ICU had a longer stay (median of 16.5 days), which was comparable to the length of hospital stay in other studies [13].

The main limitation of our study is its retrospective nature, making it difficult to establish a causative relation between exposures and outcomes. The sample size, even though being relatively large compared with other studies, prevented the performance of reliable multivariable logistic analyses to assess the predictors of ICU admission and mortality, especially since the rates of these variables were low. Being a single-center study also limits the generalizability of our findings. We also did not compare the characteristics and outcomes of patients with CAP due to HRV to those with CAP due to other causative agents, including other viruses.

In conclusion, most patients with CAP due to HRV were elderly and had significant comorbidities. Corticosteroids were used in most patients. ICU admission was required in almost one in six patients and mechanical ventilation in almost one in ten patients. The overall hospital mortality was 9.4%. Patients who were admitted to the ICU had significantly higher mortality.

## Figures and Tables

**Table 1:** Characteristics of patients admitted with rhinovirus pneumonia.

Variable	All patients *N* = 106	ICU admission *N* = 16	No ICU admission *N* = 90	*p* value
Age (years), median (Q1, Q3)	71.5 (44.0, 84.0)	69.0 (47.0, 82.8)	72.0 (43.8, 84.0)	0.81
Male gender, *N* (%)	50 (47.2)	6 (37.5)	44 (48.9)	0.40
BMI (kg/m^2^), median (Q1, Q3)	27.3 (22.7, 35.04)	30.3 (23.0, 40.0)	27.0 (22.7, 34.3)	0.27
Smoking, *N* (%)	8 (7.5)	2 (12.5)	6 (6.7)	0.42
Comorbidities, *N* (%)				
Diabetes	53 (50.0)	8 (50.0)	8 (50.0)	8 (50.0)
Hypertension	62 (59.0)	11 (68.8)	51 (57.3)	0.39
Hypothyroidism	16 (15.1)	3 (18.8)	13 (14.4)	0.66
Heart failure	37 (34.9)	8 (50.0)	29 (32.2)	0.17
Cerebrovascular accident	20 (18.9)	2 (12.5)	18 (20.0)	0.48
Chronic kidney disease	22 (20.8)	2 (12.5)	20 (22.2)	0.38
Dialysis	8 (7.5)	0 (0)	8 (8.9)	0.22
Ischemic heart disease	22 (20.8)	6 (37.5)	16 (17.8)	0.07
Dyslipidemia	27 (25.5)	3 (18.8)	24 (26.7)	0.50
Chronic respiratory disease	47 (44.3)	10 (62.5)	37 (41.1)	0.40
Liver disease	3 (2.8)	1 (6.3)	2 (2.2)	0.37
Immunosuppression	9 (8.5)	3 (18.8)	6 (6.7)	0.11
Malignancy	6 (5.7)	2 (12.5)	4 (4.4)	0.20
Transplant	3 (2.8)	1 (6.3)	2 (2.2)	0.37
Bedbound before hospitalization, *N* (%)	28 (26.4)	2 (12.5)	26 (28.9)	0.17
Presenting symptoms, *N* (%)				
Fever	41 (38.7)	5 (31.3)	36 (40.0)	0.51
RR > 30/min	34 (32.1)	10 (62.5)	24 (26.7)	0.005
Systolic BP < 90 mm Hg	9 (8.5)	3 (18.8)	6 (6.7)	0.11
*T* < 35 or >39.9°C	11 (10.4)	0 (0)	11 (12.2)	0.14
Pulse >125/min	20 (18.9)	5 (31.3)	15 (16.7)	0.17
Shortness of breath	96 (90.6)	16 (100)	80 (88.9)	0.16
Dry cough	25 (23.6)	5 (31.3)	20 (22.2)	0.43
Purulent cough	66 (62.2)	7 (43.8)	59 (65.5)	0.24
Chest pain	16 (15.1)	2 (12.5)	14 (15.6)	0.75
Myalgia/arthralgia	23 (21.7)	1 (6.3)	22 (24.4)	0.10
Fatigue	27 (25.5)	3 (18.8)	24 (26.7)	0.50
Headache	11 (10.4)	0 (0)	11 (12.2)	0.14
Hemoptysis	2 (1.9)	2 (12.5)	0 (0)	0.001
Pneumonia severity index^*∗*^, median (Q1, Q3)	107.0 (78.5, 129.8)	116.5 (91.0, 142.5)	105.0 (73.3, 128.8)	0.29
Laboratory findings, median (Q1, Q3)	10.95 (7.92, 14.13)	11.30 (8.32, 14.58)	10.95 (7.90, 14.10)	0.58
White blood cell (10^9^/L)	74.4 (62.0, 81.90)	86.4 (77.0, 91.7)	74.0 (61.03, 80.0)	0.02
Neutrophil %	13.1 (9.28, 22.60)	5.7 (3.40, 9.70)	13.9 (10.0, 24.5)	0.01
Lymphocyte %	0.37 (0.32, 0.42)	0.31 (0.26, 0.39)	0.38 (0.33, 0.43)	0.006
Hematocrit	30.1 (26.8, 32.1)	30.9 (28.9, 34.0)	30.0 (26.7, 33.0)	0.19
PTT (seconds)	1.1 (1.1, 1.3)	1.2 (1.1, 1.5)	1.1 (1.1, 1.2)	0.11
INR	38 (35.8)	4 (25.0)	34 (37.8)	0.33
BUN >11 mmol/L, *N* (%)	136 (133, 139)	136 (135, 137.75)	136 (133, 139)	0.82
Sodium (meq/L)	88.0 (65.0, 136.0)	87.0 (83.3, 135.8)	88.0 (64.0, 136.0)	0.76
Creatinine (*μ*mol/L)	1.7 (1.3, 2.7)	2.0 (1.6, 4.2)	1.6 (1.2, 2.6)	0.47
Lactic acid (mmol/L) pH	7.36 (7.30, 7.40)	7.38 (7.34, 7.44)	7.35 (7.30, 7.40)	0.14
PaO_2_ (mm Hg)	73.0 (61.2, 82.0)	78.3 (62.0, 122.3)	70.6 (59.0, 79.6)	0.12
Leukocytosis (white blood cell> 15.00 10^9^/L, *N* (%)	20 (18.9)	3 (18.8)	17 (18.9)	1.0
Lymphopenia ^*∗∗*^ (<1000 × 10^6^/L), *N* (%)	26/87 (29.9)	5/7 (71.4)	21/80 (26.3)	0.01
Neutrophil-to-lymphocyte ratio, median (Q1, Q3)	5.9 (3.1, 8.4)	10.4 (6.0, 18.5)	5.2 (2.7, 8.0)	0.02
C-reactive protein (mg/L), median (Q1, Q3)^*∗*^	42.5 (23.0, 108.0)	30.2 (9.0, 72.0)	49.5 (23.0, 116.5)	0.38
C-reactive protein> 40 mg/L, *N* (%)	24/45 (53.3)	3/7 (42.9)	21/38 (55.3)	0.69
Procalcitonin (ng/ml), median (Q1, Q3)^*∗*^	0.10 (0.05, 0.56)	0.41 (0.12, 0.58)	0.08 (0.04, 0.56)	0.35
Procalcitonin> 0.5 ng/ml, *N* (%)	9/31 (29.0)	2/4 (50.0)	7/27 (25.9)	0.56
Bacterial coinfection, *N* (%)	7 (6.6)	6 (6.7)	1 (6.3)	1.0
Chest X-ray findings^*∗∗*^				
Unilateral infiltrates	26/101 (25.7)	5/16 (31.3)	21/85 (24.7)	0.55
Bilateral infiltrates	47/101 (46/5)	11/16 (68.8)	36/85 (42.4)	0.06
Unilateral pleural effusion	22/101 (21.8)	3/16 (18.8)	19/85 (22.4)	1.0
Bilateral pleural effusion	16/101 (15.8)	3/16 (18.8)	13/85 (15.3)	0.71
Chest computed tomography findings ^*∗∗*^				
Unilateral infiltrates	6/20 (30)	2/6 (33)	4/14 (28.6)	1.0
Bilateral infiltrates	7/20 (35)	3/6 (50)	4/14 (28.6)	0.61
Unilateral pleural effusion	2/20 (10)	1/6 (16.7)	1/14 (7.1)	0.52
Bilateral pleural effusion	4/20 (20)	2/6 (33.3)	2/14 (14.3)	0.55

BMI: body mass index, BP: blood pressure, BUN: blood urea nitrogen, ICU: intensive care unit, INR: international normalized ratio PTT: partial thromboplastin time, Q1: first quartile, Q3: third quartile, RR: respiratory rate, and T: body temperature. ^*∗*^Complete data to calculate PSI was available in only 42 patients. C-reactive protein level within 2 days of admission was available in 45 patients. Procalcitonin level within 2 days of admission was available in 31 patients. ^*∗∗*^Data were missing for some patients. The denominator represents the number of patients with available data.

**Table 2:** Management of patients.

Variable	All patients *N* = 106	ICU admission *N* = 16	No ICU admission *N* = 90	*p* value
Mechanical ventilation, *N* (%)	9 (8.7)	9 (69.2)	0 (0)	<0.001
Vasopressors, *N* (%)	6 (5.7)	6 (37.5)	0 (0)	<0.001
Use of steroids, *N* (%)	58 (54.7)	10 (62.5)	48 (53.3)	0.50
Initial daily dose (mg of prednisone or equivalent), median (Q1, Q3)	120 (40, 120)	50 (40, 105)	120 (50, 120)	0.02
Antimicrobial therapy, *N* (%)				
No initial antibiotic	7 (6.7)	0 (0)	7 (8)	0.60
Fluoroquinolones	4 (3.8)	0 (0)	4 (4.5)	1.0
Beta lactam alone	13 (12.3)	1 (6.3)	12 (13.3)	0.69
Antipseudomonal beta lactam	7 (6.6)	2 (12.5)	5 (5.6)	0.28
Beta lactam + azithromycin	43 (40.6)	3 (18.8)	40 (44.4)	0.06
Antipseudomonal beta lactam + azithromycin	30 (28.3)	10 (62.5)	20 (22.2)	0.002
Oseltamivir, *N* (%)	73/106 (68.9)	10 (62.5)	63/90 (70)	0.55

ICU: intensive care unit, Q1: first quartile, and Q3: third quartile.

**Table 3:** Outcomes of patients.

Variable	All patients *N* = 106	ICU admission *N* = 16	No ICU admission *N* = 90	*p* value
MV duration (days), median (Q1, Q3)	8.00 (5.50, 12.50)	8.0 (5.5, 12.5)	0	-
LOS in ICU (days), median (Q1, Q3)	10.0 (5.0, 17.0)	10.0 (5.0, 17.0)	0	-
LOS in hospital (days), median (Q1, Q3)	5.0 (3.00, 8.00)	16.50 (8.8, 25.5)	5.0 (3.0, 7.00)	<0.001
Tracheostomy, *N* (%)	1/83 (1.2)	1/14 (7.1)	0/69 (0)	0.03
Hospital mortality, *N* (%)	10 (9.4)	6 (37.5)	4 (4.4)	<0.001

ICU: intensive care unit, LOS: length of stay, MV: mechanical ventilation, Q1: first quartile, and Q3: third quartile.

## Data Availability

Data are available upon reasonable request from the corresponding author.
